# Ancient Mitochondrial Capture as Factor Promoting Mitonuclear Discordance in Freshwater Fishes: A Case Study in the Genus *Squalius* (Actinopterygii, Cyprinidae) in Greece

**DOI:** 10.1371/journal.pone.0166292

**Published:** 2016-12-01

**Authors:** Silvia Perea, Jasna Vukić, Radek Šanda, Ignacio Doadrio

**Affiliations:** 1 Biodiversity and Evolutionary Group, Museo Nacional de Ciencias Naturales-CSIC, C/José Gutiérrez Abascal, Madrid, Spain; 2 Department of Ecology, Faculty of Science, Charles University, Viničná, Prague, Czech Republic; 3 Department of Zoology, National Museum, Václavské nám, Prague, Czech Republic; University of California Santa Cruz, UNITED STATES

## Abstract

Hybridization and incomplete lineage sorting are common confounding factors in phylogeny and speciation resulting in mitonuclear disparity. Mitochondrial introgression, a particular case of hybridization, may, in extreme cases, lead to replacement of the mitochondrial genome of one species with that of another (mitochondrial capture). We investigated mitochondrial introgression involving two species of the cyprinid genus *Squalius* in the western Peloponnese region of Greece using molecular and morphological data. We found evidence of complete mitochondrial introgression of *Squalius keadicus* into two populations recognized as *Squalius peloponensis* from the Miras and Pamissos River basins and a divergence of mitochondrial genomes of *S*. *keadicus* from the Evrotas basin from that of the introgressed populations dating from the Pleistocene. Secondary contact among basins is a possible factor in connection of the species and the introgression event. Morphological analyses support the hypothesis of mitochondrial introgression, as *S*. *keadicus* was different from the other three populations recognized as *S*. *peloponensis*, although significant differences were found among the four populations. Isolation by geographical barriers arose during Pleistocene in the western Peloponnese were the source of the evolution of the two reciprocally monophyletic subclades found in the *S*. *keadicus* mitochondrial clade, and the morphological differences found among the four populations. Along with the lack of structure in the nuclear genome in the three populations ascribed to *S*. *peloponensis*, this suggests an incipient speciation process occurring in these *Squalius* species in the western Peloponnese.

## Introduction

The use of mitochondrial DNA for inferring phylogenetic or phylogeographic relationships has been traditionally preferred over the use of the nuclear genome, as mtDNA accurately reflects recent divergence patterns than do nuclear markers [1]. Nevertheless, concordant patterns between mtDNA and nuclear DNA are not always observed in nature [2–4].

Evolutionary processes such as hybridization and incomplete lineage sorting are common confounding factors in phylogeny and speciation and primary causes of mitonuclear discordance [3–5]. Incomplete lineage sorting occurs when the coalescence time of genes and speciation differ, i.e. when lineages fail to sort out at the same time at speciation happens [6]. Therefore, gene trees do not always represent the true relationships among taxa because different genes may have not branched at the same time [7–9]. Distinguishing incomplete lineage sorting from hybridization can be difficult as these two processes may originate the same phylogenetic tree [10–12]. Nonetheless, among others, any biogeographic pattern is expected when incomplete lineage sorting is the confounding factor [4].

Hybridization between species involves mating between unrelated organisms regardless the taxonomic status and, in some cases, may lead to gene transfer, a common process in plants [13], or to very complex evolutionary processes occurring in freshwater fishes [14–15]. Introgression is a particular case of hybridization and occurs when gene flow exists between populations that hybridize and hybrids backcross to one or both parental populations [16]. Within this framework, mitochondrial capture is defined as complete mitochondrial introgression, a situation in which the mitochondrial genome of one species is replaced with that of another in an entire population, as a consequence of selective backcrossing of hybrids with one of the parent species [2–3, 17–19]. In hybridization, the complete replacement of the mitochondrial DNA of one species with that of another is more common than is replacement of a portion of the nuclear genome, due to maternal inheritance and the four-fold smaller effective population size of mitochondrial DNA compared to nuclear DNA, implying that mtDNA will complete the process of lineage sorting more rapidly than will nuclear DNA [1, 3, 20–21]. Understanding evolutionary processes such as hybridization, mitochondrial introgression, and incomplete lineage sorting is of vital importance to inference reliable phylogenies and reconstruction of evolutionary history and speciation processes, especially in currently allopatric species. Revealing mitochondrial introgression may be the only way to identify past hybridization of allopatric species [17].

In general, mitonuclear discordance due to mitochondrial introgression occurs more frequently in certain taxonomic groups, including mammals and fish; whereas, in groups such as birds and butterflies, in which the female is heterogametic, mitochondrial introgression is usually reduced, conforming to Haldane’s rule [4]. Examples of the complete replacement of mitochondrial DNA are found in various taxa [18–19, 22–25], including freshwater fishes [26–28].

**Fig 1:**
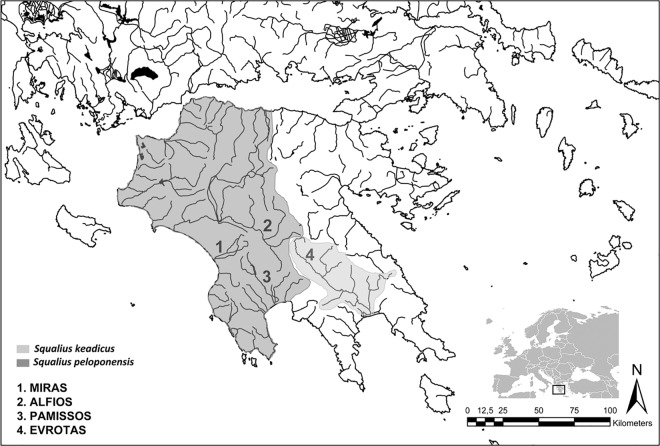
Locality samples and distribution range of *S*. *keadicus* and *S*. *peloponensis* according to [37].

Within freshwater fishes the preponderance of hybridization is greater in cyprinids than is in other freshwater fish families such as salmonids or cichlids and several examples are observed [29–32]. A clear example of mitonuclear discordance has been described for the genus *Squalius* in the Greek Peloponnese. Previous studies focusing on a more general framework of phylogenetic relationships of *Squalius* hypothesised the presence of mitochondrial introgression to explain discrepancy found in the mitochondrial (DNA sequences) and nuclear (allozyme) phylogenetic position of *S*. *keadicus* (Stephanidis, 1971) and *S*. *peloponensis* (Valenncienes, 1844) [33–34]. These two species, currently showing allopatric distribution, belong to two different evolutionary lineages recognized within the genus *Squalius*; *S*. *peloponesis* belongs to the Euroasiatic group while *S*. *keadicus* is included together with other small-sized species within the Mediterranean group. These two evolutionary lineages have been isolated since the Middle Miocene [35–36]. *Squalius keadicus* is a small- to medium-sized fish endemic to the Evrotas Basin in the southwestern Peloponnese. *Squalius peloponensis* is a medium to large fish and has a broader distribution range in the western Peloponnese, encompassing several hydrological basins (Fig 1) [37]. The morphological distinction between both species is clear and several diagnostic morphometric and meristic characters are identified to distinguishing them from other Balkan *Squalius* species. Head length is 24–27% standard length in *S*. *peloponensis*. The posterior margin of the anal fin is different in both species, being straight in *S*. *keadicus* and slightly convex in *S*. *peloponensis*. Meristic diagnostic characters of *S*. *keadicus* show 44–49 scales on lateral line whereas in *S*. *peloponensis* this range is 40–44. The colouration of both species also differs; *S*. *keadicus* shows in live a conspicuous blackish stripe on flank from eye to caudal fin base and very dark scales in upper half of flank, which are not present in *S*. *peloponensis* [37].

With regards to the paleogeographical context in which the two species have evolved, the southwestern Peloponnese lies near the Hellenic Trench in the Eastern Mediterranean region, one of the most active areas in the Euroasiatic-African convergence zone owing to the northward subduction of the African plate beneath the Aegean plate [38]. The region has a complex tectonic history and numerous currently active northwestern-southeastern tectonic faults [38–41]. The formation of the hydrological network of the Greek Peloponnese was complex, characterized by Late Miocene-Quaternary compressional structures as well as Pliocene lacustrine-marine and Pleistocene terrestrial-fluvial sedimentary successions leading to the formation of fluvial terraces [38, 42–43]. Such paleogeographic complexity may have promoted ancient contact among freshwater fauna inhabiting the basins of the western Peloponnese, resulting in hybridization events.

Within the framework of mitonuclear disparity, the aim of this study was to investigate the mitochondrial capture between two species of *Squalius* from the Greek Peloponnese using molecular and morphological analyses of three populations of *S*. *peloponensis* and one of *S*. *keadicus*. We also establish a temporal and biogeographical framework for the introgression episode.

## Material and Methods

### Sampling

Specimens of four populations of *Squalius* were collected by electrofishing from basins in the western Peloponnese region of Greece following the European regulations (*EN ISO 14011*:*200*. *Water quality—Sampling of fish with electrophising*) (Table 1; Fig 1). Sampling was performed with authorised permission from the Greek Ministry of Environment, Energy and Climatic Change and after consulting the Animal Care and Use Committee of the National Museum of Prague (č.j.NM/2014/258). Immediately after capture, all individuals were anesthetized using Tricaine methanesulfonate (MS-222) to alleviate suffering. A piece of ventral fin sample (~3 mm^2^ fin clips) was obtained from 61 fish belonging to the four Greek populations analysed (nine to *S*. *keadicus* and 52 to *S*. *peloponensis*). All tissues were preserved in 95% ethanol and stored at 4°C until is processing at laboratory for molecular analyses. These 61 specimens analysed for molecular markers were sacrificed for morphological analyses following humanely euthanization with overdose of MS-222 according to European Commission regulations (Directive 2010/63/EU). Death was confirmed after no gill movement was observed for at least 10 minutes. These new sampled specimens used for morphological purposes were preserved in a solution of 5% formalin and after a week they were transferred to a 70% ethanol solution. These new-collected specimens have been deposited in the Fish Collection of the National Museum of Prague (Czech Republic). The remaining specimens used for morphological analyses in this study came from historical deposits of the Fish Collections of the Museo Nacional de Ciencias Naturales, CSIC, in Madrid (Spain). Voucher tissues of new collected specimens are deposited in the DNA Collections of the Museo Nacional de Ciencias Naturales in Madrid, CSIC, (Spain) and in the National Museum of Prague (Czech Republic).

**Table 1:** Specimens used in molecular and morphological analyses and their voucher numbers. GenBank accession numbers for all analysed genes and labels in phylogenetic trees. All these specimens except those marked with an * symbol, proceeding from GenBank database, have been used in morphological analyses.

Species/Population (Voucher number)	Geographic coordinates	*MT-CYB*	*RAG1* (phased alleles)	*S7* (phased alleles)	Label in phylogenetic trees
*Squalius keadicus/Evrotas* (AJ252820)*	-	AJ252820	-	-	*Squalius keadicus* 1
*Squalius keadicus/Evrotas* (AF090760)*	36° 51' 15.6" N, 22° 40' 29.8" E	AF090760	-	-	*Squalius keadicus* 2
*Squalius keadicus/Evrotas* (MNCN_ICTIO 123.875)	36° 51' 15.6" N, 22° 40' 29.8" E	HM560185	HM560583	HM560572	*Squalius keadicus* 3
*Squalius keadicus/Evrotas* (NMP PV6 F1556)	37° 5' 28.71" N, 22°25'38.74" E	KY070419	KY070433 (a); KY070434 (b)	KY070547 (a); KY070548 (b)	*Squalius keadicus* 4
*Squalius keadicus/Evrotas* (NMP PV6 F1557)	37° 5' 28.71" N, 22°25'38.74" E	KY070420	KY070435 (a); KY070436 (b)	KY070549 (a); KY070550 (b)	*Squalius keadicus* 5
*Squalius keadicus/Evrotas* (NMP PV6 F1566)	37° 5' 28.71" N, 22°25'38.74" E	KY070421	KY070425 (a); KY070426 (b)	KY070503 (a); KY070504 (b)	*Squalius keadicus* 6
*Squalius keadicus/Evrotas* (NMP PV6 F1567)	37° 5' 28.71" N, 22°25'38.74" E	KY070422	KY070427 (a); KY070428 (b)	KY070505 (a); KY070506 (b)	*Squalius keadicus* 7
*Squalius keadicus/Evrotas* (NMP PV6 F1570)	37° 5' 28.71" N, 22°25'38.74" E	KY070423	KY070429 (a); KY070430 (b)	KY070551 (a); KY070552 (b)	*Squalius keadicus* 8
*Squalius keadicus/Evrotas* (NMP PV6 F1571)	37° 5' 28.71" N, 22°25'38.74" E	KY070424	KY070431 (a); KY070432 (b)	KY070553 (a); KY070554 (b)	*Squalius keadicus* 9
*Squalius peloponensis/Alfios* (MNCN_ICTIO 120.548)	37° 28' 21.7" N, 22° 04' 33.9" E	KY070368	-	KY070515 (a); KY070516 (b)	Alfios 1
*Squalius peloponensis/Alfios* (MNCN_ICTIO 120.559)	37° 28' 21.7" N, 22° 04' 33.9" E	KY070372	KY070497 (a); KY070498 (b)	-	Alfios 2
*Squalius peloponensis/Alfios* (MNCN_ICTIO 120.563)	37° 28' 21.7" N, 22° 04' 33.9" E	KY070374	KY070501 (a); KY070502 (b)	KY070523 (a); KY070524 (b)	Alfios 3
*Squalius peloponensis/Alfios* (MNCN_ICTIO 120.553)	37° 28' 21.7" N, 22° 04' 33.9" E	KY070369	KY070491 (a); KY070492 (b)	KY070517 (a); KY070518 (b)	Alfios 4
*Squalius peloponensis/Alfios* (MNCN_ICTIO 120.555)	37° 28' 21.7" N, 22° 04' 33.9" E	KY070370	-	-	Alfios 5
*Squalius peloponensis/Alfios* (MNCN_ICTIO 120.556)	37° 28' 21.7" N, 22° 04' 33.9" E	KY070371	KY070493(a); KY070494 (b)	KY070519 (a); KY070520 (b)	Alfios 6
*Squalius peloponensis/Alfios* (MNCN_ICTIO 120.560)	37° 28' 21.7" N, 22° 04' 33.9" E	KY07037	KY070499 (a); KY070500 (b)	KY070521 (a); KY070522 (b)	Alfios 7
*Squalius peloponensis/Alfios* (MNCN_ICTIO 120.557)	37° 28' 21.7" N, 22° 04' 33.9" E	-	KY070495 (a); KY070496 (b)	-	Alfios 8
*Squalius peloponensis/Miras* (NMP PV6 F1470)	37° 17' 34.2" N, 21° 42' 7.86" E	KY070407	KY070467 (a); KY070468 (b)	KY070525 (a); KY070526 (b)	Miras 1
*Squalius peloponensis/Miras* (NMP PV6 F1471)	37° 17' 34.2" N, 21° 42' 7.86" E	KY070408	KY070469 (a); KY070470 (b)	KY070527 (a); KY070528 (b)	Miras 2
*Squalius peloponensis/Miras* (NMP PV6 F1472)	37° 17' 34.2" N, 21° 42' 7.86" E	KY070409	KY070471 (a); KY070472 (b)	-	Miras 3
*Squalius peloponensis/Miras* (NMP PV6 G210)	37° 17' 34.2" N, 21° 42' 7.86" E	KY070410	KY070473 (a); KY070474 (b)	KY070555 (a); KY070556 (b)	Miras 4
*Squalius peloponensis/Miras* (NMP PV6 G211)	37° 17' 34.2" N, 21° 42' 7.86" E	KY070411	KY070475 (a); KY070476 (b)	KY070557 (a); KY070558 (b)	Miras 5
*Squalius peloponensis/Miras* (NMP PV6 G212)	37° 17' 34.2" N, 21° 42' 7.86" E	KY070412	KY070477 (a); KY070478 (b)	KY070559 (a); KY070560 (b)	Miras 6
*Squalius peloponensis/Miras* (NMP PV6 G213)	37° 17' 34.2" N, 21° 42' 7.86" E	KY070413	KY070479 (a); KY070480 (b)	KY070561 (a); KY070562 (b)	Miras 7
*Squalius peloponensis/Miras* (NMP PV6 G214)	37° 17' 34.2" N, 21° 42' 7.86" E	KY070414	KY070481 (a); KY070482 (b)	KY070563 (a); KY070564 (b)	Miras 8
*Squalius peloponensis/Miras* (NMP PV6 G215)	37° 17' 34.2" N, 21° 42' 7.86" E	KY070415	KY070483 (a); KY070484 (b)	KY070565 (a); KY070566 (b)	Miras 9
*Squalius peloponensis/Miras* (NMP PV6 G219)	37° 17' 34.2" N, 21° 42' 7.86" E	KY070416	KY070485 (a); KY070486 (b)	KY070567 (a); KY070568 (b)	Miras 10
*Squalius peloponensis/Miras* (NMP PV6 G220)	37° 17' 34.2" N, 21° 42' 7.86" E	KY070417	KY070487 (a); KY070488 (b)	KY070569 (a); KY070570 (b)	Miras 11
*Squalius peloponensis/Miras* (NMP PV6 G222)	37° 17' 34.2" N, 21° 42' 7.86" E	KY070418	KY070489 (a); KY070490 (b)	KY070571 (a); KY070572 (b)	Miras 12
*Squalius peloponensis/Miras* (MNCN_ ICTIO 94.744)	37° 00' 00.0" N, 21° 44' 23.5" E	KY070404	KY070461 (a); KY070462 (b)	KY070507 (a); KY070508 (b)	Miras 13
*Squalius peloponensis/Miras* (MNCN_ICTIO 94.745)	37° 00' 00.0" N, 21° 44' 23.5" E	KY070405	KY070463 (a); KY070464 (b)	KY070509 (a); KY070510 (b)	Miras 14
*Squalius peloponensis/Miras* (MNCN_ICTIO 94.746)	37° 00' 00.0" N, 21° 44' 23.5" E	KY070406	KY070465 (a); KY070466 (b)	KY070511 (a); KY070512 (b)	Miras 15
*Squalius peloponensis/Pamissos* (NMP PV6 F1514)	37° 9'25.21" N, 21°58'43.16" E	KY070387	-	-	Pamissos 1
*Squalius peloponensis/Pamissos* (NMP PV6 F1515)	37° 9'25.21" N, 21°58'43.16" E	KY070388	-	-	Pamissos 2
*Squalius peloponensis/Pamissos* (NMP PV6 F1516)	37° 9'25.21" N, 21°58'43.16" E	KY070389	-	-	Pamissos 3
*Squalius peloponensis/Pamissos* (NMP PV6 F1517)	37° 9'25.21" N, 21°58'43.16" E	KY070390	-	-	Pamissos 4
*Squalius peloponensis/Pamissos* (NMP PV6 F1518)	37° 9'25.21" N, 21°58'43.16" E	KY070391	-	-	Pamissos 5
*Squalius peloponensis/Pamissos* (NMP PV6 F1519)	37° 9'25.21" N, 21°58'43.16" E	KY070392	KY070437 (a); KY070438 (b)	KY070529 (a); KY070530 (b)	Pamissos 6
*Squalius peloponensis/Pamissos* (NMP PV6 F1520)	37° 9'25.21" N, 21°58'43.16" E	KY070393	KY070439 (a); KY070440 (b)	KY070531 (a); KY070532 (b)	Pamissos 7
*Squalius peloponensis/Pamissos* (NMP PV6 F1521)	37° 9'25.21" N, 21°58'43.16" E	KY070394	KY070441 (a); KY070442 (b)	-	Pamissos 8
*Squalius peloponensis/Pamissos* (NMP PV6 F1522)	37° 9'25.21" N, 21°58'43.16" E	KY070395	KY070443 (a); KY070444 (b)	KY070533 (a); KY070534 (b)	Pamissos 9
*Squalius peloponensis/Pamissos* (NMP PV6 F1523)	37° 9'25.21" N, 21°58'43.16" E	KY070396	KY070445 (a); KY070446 (b)	KY070535 (a); KY070536 (b)	Pamissos 10
*Squalius peloponensis/Pamissos* (NMP PV6 F1524)	37° 9'25.21" N, 21°58'43.16" E	KY070397	KY070447 (a); KY070448 (b)	KY070535 (a); KY070536 (b)	Pamissos 11
*Squalius peloponensis/Pamissos* (NMP PV6 F1525)	37° 9'25.21" N, 21°58'43.16" E	KY070398	KY070449 (a); KY070450 (b)	KY070537 (a); KY070538 (b)	Pamissos 12
*Squalius peloponensis/Pamissos* (NMP PV6 F1526)	37° 9'25.21" N, 21°58'43.16" E	KY070399	KY070451 (a); KY070452 (b)	KY070539 (a); KY070540 (b)	Pamissos 13
*Squalius peloponensis/Pamissos* (NMP PV6 F1527)	37° 9'25.21" N, 21°58'43.16" E	KY070400	KY070433 (a); KY070454 (b)	KY070541 (a); KY070542 (b)	Pamissos 14
*Squalius peloponensis/Pamissos* (NMP PV6 F1541)	37° 9'25.21" N, 21°58'43.16" E	KY070401	KY070455 (a); KY070456 (b)	KY070543 (a); KY070544 (b)	Pamissos 15
*Squalius peloponensis/Pamissos* (G244)	37°15'17.39"N, 21°53'45.15" E	KY070402	KY070457 (a); KY070458 (b)	KY070545 (a); KY070546 (b)	Pamissos 16
*Squalius peloponensis/Pamissos* (G245)	37°15'17.39"N, 21°53'45.15" E	KY070403	KY070459 (a); KY070460 (b)	KY070573 (a); KY070574 (b)	Pamissos 17

The study included the Evrotas River population, this being the type locality of *S*. *keadicus* and currently the only known locality for the species, and three populations recognized as *S*. *peloponensis* from the Alfios, Miras, and Pamissos basins (Fig 1). The type locality of *S*. *peloponensis* is uncertain, and only the Peloponnese peninsula (= Morea) is cited in its description [44]. Additional species of *Squalius* along its distribution range were included in the molecular analyses to assess the phylogenetic position of the four investigated populations (Table A in S1 File). The genus *Petroleuciscus* was used as the outgroup based on previous phylogenetic studies [35–36].

### DNA extraction, amplification and sequencing

Eight to 17 specimens per population were analysed (Table 1). Total genomic DNA was isolated using the commercial kit Biosprint 15 for blood and tissue (Qiagen). For each specimen the complete mitochondrial cytochrome *b* (*MT-CYB*; 1140bp) gene and two nuclear genes, the first intron of the ribosomal *S7* gene (*S7*; final alignment including gaps = 977bp) and the third exon of the recombination activating protein gene (*RAG-1*; 1473bp), were amplified. The PCR protocols and primers followed [36]. The PCR products were purified by Exo-SAP-IT (USB, Cleveland, OH, USA) and directly sequenced by Macrogen Europe (Amsterdam, The Netherlands; http://www.macrogen.com) using a 3730XL DNA sequencer. All new sequences of haplotypes and alleles obtained in this study were deposited in the GenBank database (Accession Numbers: *MT-CYB*: KY070368-KY070424; *RAG1*: KY070425-KY070574; *S7*: KY070503-KY070574).

### Phylogenetic analyses

For the nuclear markers, phylogenetic inference of independent alleles was also conducted on gamete phases using the PHASE algorithm [45–46] implemented in DnaSP v. 5.0. [47] with a probability threshold of 0.9 to resolve alleles. Sequences were aligned using the default pairwise and multiple alignment parameters in Clustal W [48] implemented in MEGA v.7 [49]. Alignments were later revised. Recombination of nuclear genes was assessed by the PHI test [50] implemented in SplitsTree v. 4.13 [51]. No traces of recombination were found in either *RAG-1* or *S7* genes (*p* = 1.0 for both genes).

For phylogenetic analyses, the best-fit model of evolution for each independent gene was estimated using jModelTest v2 [52] (Table B in S1 File). Model parameters were used for subsequent phylogenetic analyses. Phylogenies based on Bayesian inference (BI) and maximum likelihood (ML) were constructed for independent genes in order to assess the phylogenetic position of each population based on mitochondrial and nuclear markers. BI was performed in MrBayes 3.2 [53]. Two simultaneous analyses were run for 10 million generations, each with four MCMC chains sampling every 1000 generations. Convergence was assessed using Tracer v.1.6 [54]. The 50% majority rule consensus tree was constructed after discarding the first 10% of generations as burn-in. For ML analyses, we used RaxML software implemented in the Trex-online server [55], employing the substitution model GTR+G+I for *MT-CYB* and GTR+G for nuclear genes and the rapid bootstrapping algorithm to estimate node confidence using different random seeds (1000 replicates) [56].

Uncorrected-*p* genetic distances between mitochondrial haplotypes and between nuclear phased alleles were estimated using the MEGA v.7 software [49].

### Molecular clock

To establish a temporal framework for the introgression event, we estimated divergence time among the populations of *Squalius keadicus* (Evrotas R.) and the non-introgressed and introgressed populations of *S*. *peloponensis* using an uncorrelated relaxed molecular clock based on the *MT-CYB* gene implemented in BEAST v.2.0. [57]. To incorporate a speciation-model tree prior into the analyses, the birth-death model with incomplete taxon sampling [58], we used one specimen per species to estimate divergence time. For *S*. *peloponensis*, we used a specimen from the Alfios River (non-introgressed population) and one from the Miras River (introgressed population). As in the phylogenetic analysis, several species of the genus *Petroleuciscus* were used as outgroup. We calibrated the molecular clock using two fossil species of the genus *Squalius* (formerly within the genus *Leuciscus*) as lognormal prior: *Leuciscus antunesi* Gaudant 1977, found in a Portuguese deposit of the Middle Miocene period (13.5–14.5 Ma; [59–60]) and *Leuciscus aff*. *cephalus* from near the Aliakmon River in Greece (Lava 2 deposit) from Upper Miocene (6.56 Ma) [61]. The calibration point based on *L*. *antunesi* was allocated in the stem group of the Iberian *Squalius* species belonging to the Mediterranean lineage, whereas the age of the fossil *Leuciscus aff*. *cephalus* was placed in the crown of the current *Squalius cephalus* group. The MCMC analyses were run for 100 million generations, with parameters logged every 10000 generations. The remaining parameters were default parameters of the software. Convergence of parameters was evaluated using Tracer v.1.6 [54] and results were summarized in TreeAnnotator v.2.0 [57].

### Morphological analyses

**Fig 2:**
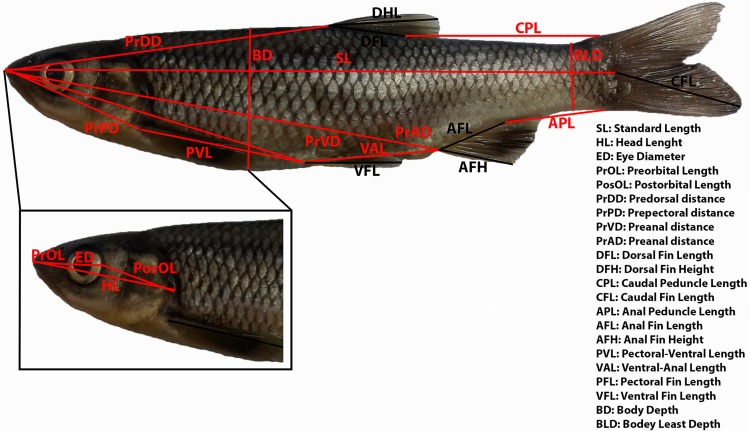
Morphometric variables measured in the *Squalius* populations analysed.

Morphological analyses included specimens from the Alfios, Miras, Pamissos and Evrotas basins. For each specimen, 23 morphometric and five meristic variables were recorded. All morphometric measurements were performed using digital callipers. Measurements and counts followed [62] and included standard length (SL), head length (HL), eye diameter (ED), interorbital width (IW), preorbital length (PrOL), postorbital length (PosOL), pre-dorsal distance (PrDD), pre-pectoral distance (PrPD), pre-ventral distance (PrPV), pre-anal distance (PrPA), caudal peduncle length (CPL), anal peduncle length (APL), pectoral-ventral length (PVL), ventral-anal length (VAL), body depth (BD), body least depth (BLD), dorsal fin height (DFH), dorsal fin length (DFL), anal fin height (AFH), anal fin length (AFL), pectoral fin length (PFL), ventral fin length (VFL), caudal fin length (CFL), lateral line scales (LLS), upper transverse scales (UTS), lower transverse scales (LTS), dorsal fin rays (D) and anal fin rays (A) (Fig 2).

After constructing the measurement matrix, Burnaby’s method was used to correct for size effect in morphometric variables [63–64]. A canonical variate analysis (CVA) was conducted to identify the variables most explanatory of the morphological variation among the analysed populations with the aim to maximize differences among populations. Non-parametric Kruskal–Wallis and Mann–Whitney *post hoc* comparisons were used to test for significant differences of morphological variables among populations. All analyses were performed using the Burnaby’s corrected matrix. Statistical analyses were carried out in PAST v.3.12 software [65].

## Results

### Molecular analyses

**Fig 3:**
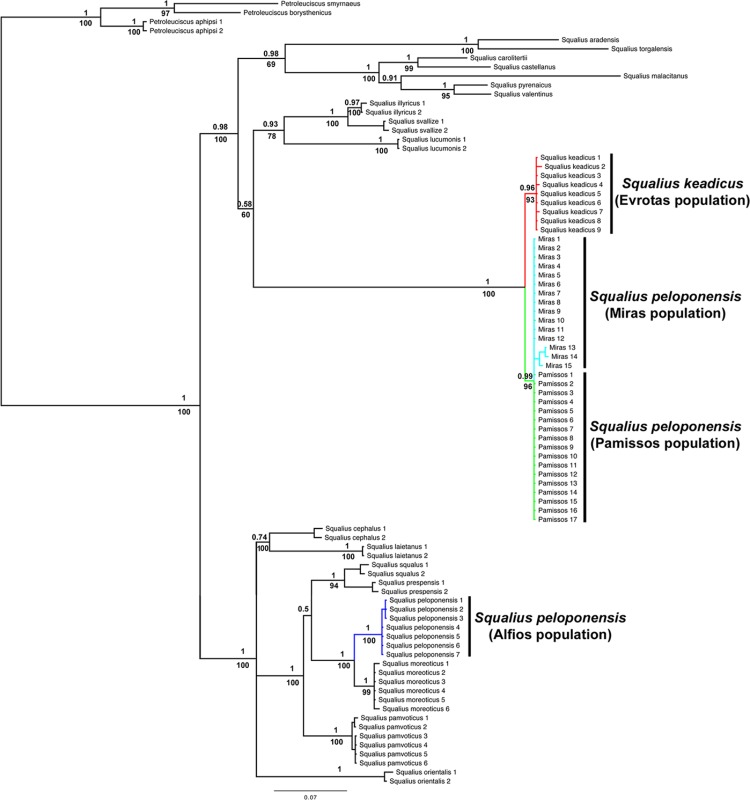
Phylogenetic tree based on the *MT-CYB* gene. Bootstrap values for ML analysis below branches. Posterior probability values for Bayesian inference above branches.

**Fig 4:**
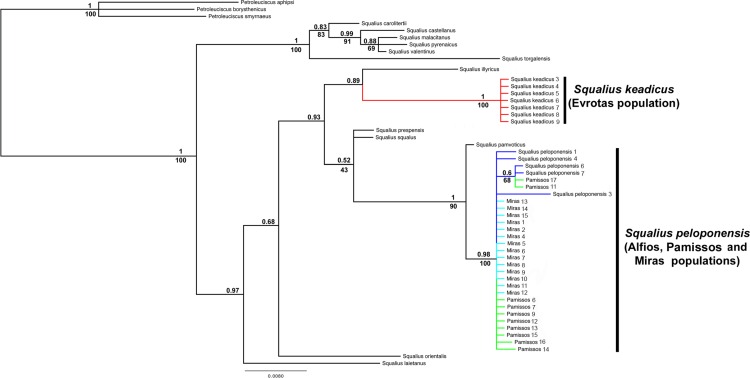
Phylogenetic tree based on the nuclear S7 gene. Bootstrap values for ML analysis below branches. Posterior probability values for Bayesian inference above branches.

**Fig 5:**
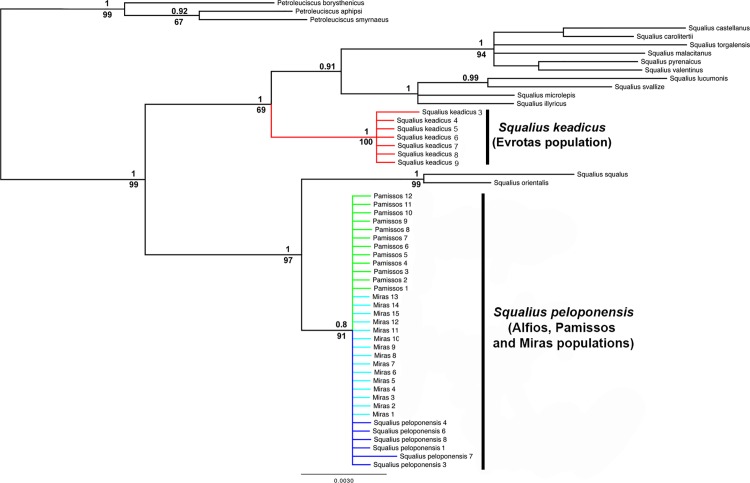
Phylogenetic tree based on the nuclear *RAG1* gene. Bootstrap values for ML analysis below branches. Posterior probability values for Bayesian inference above branches.

**Fig 6:**
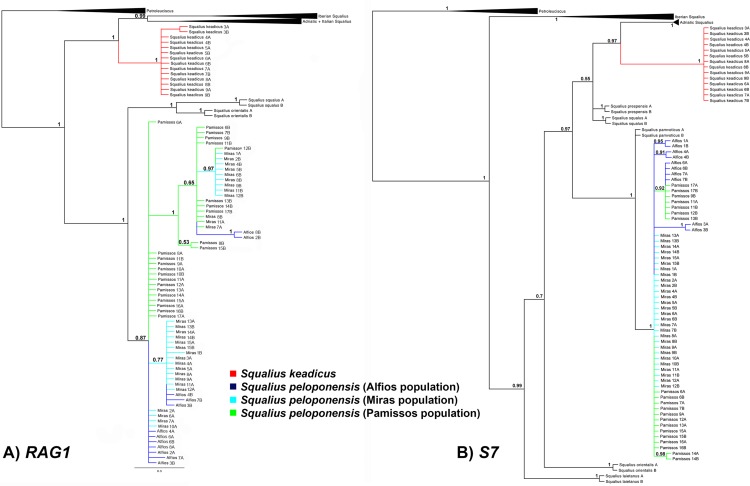
Phylogenetic tree based on the phased nuclear *RAG1* (A) and *S7* (B) genes. Posterior probability values for Bayesian inference above branches. Blue = Alfios (*S*. *peloponensis*); Red = Evrotas (*S*. *keadicus*); Green = *S*. *keadicus*-like mitochondrial (Miras and Pamissos populations).

The result of mitochondrial phylogenetic analyses is shown in Fig 3. The *Squalius* populations from the Pamissos and Miras basins clustered together, forming a monophyletic clade, sister to *S*. *keadicus*. These two clades (*S*. *keadicus* and Pamissos/Miras *S*. *peloponensis*) were highly supported as monophyletic in the BI and ML analyses. Nuclear genes yielded tree topology inconsistent with that of the mitochondrial marker, with the Pamissos and Miras populations showing a highly supported cluster with *S*. *peloponensis* from the Alfios Basin, forming a monophyletic clade (Figs 4, 5 and 6). All phased alleles for the nuclear genes *RAG1* and *S7* from the Miras and Pamissos populations were nested within the clade of *S*. *peloponensis* and none of them was grouped with *S*. *keadicus*. No evidence of genetic structure was observed in the nuclear clade comprising the Alfios, Miras, and Pamissos populations.

Uncorrected-*p* genetic distances between *S*. *keadicus* and the introgressed populations (Miras and Pamissos) for the three analysed genes are presented in Table 2. Table C in S1 File shows diagnostic autapomorphies characteristic of each population. For the *MT-CYB*, 115 autapomorphies (21 transversions) were recognized in the Alfios Basin population relative to the three other populations, five (1 transversion) were observed in *S*. *keadicus* and three in the introgressed populations, one of them present only in the Pamissos river system. For the *RAG1* gene, 13 autapomorphies (4 transversions) were found in *S*. *keadicus* in relation to the three populations recognized as *S*. *peloponensis*. For the S7 gene, 23 autapomorphies (9 transversions) appeared in *S*. *keadicus*, as well as three insertions and three deletions not found in the other three populations.

**Table 2:** Uncorrected-*p* genetic distances (%) between (below diagonal) and within (in diagonal) populations for the three genes analysed.

***MT-CYB***				
	**Evrotas**	**Pamissos**	**Miras**	**Alfios**
**Evrotas**	0.01			
**Pamissos**	0.6	0.00		
**Miras**	0.6	0.1	0.01	
**Alfios**	11.0	10.7	10.8	0.01
**S7**				
	**Evrotas**	**Pamissos**	**Miras**	**Alfios**
**Evrotas**	0.0			
**Pamissos**	2.8	0.1		
**Miras**	2.7	0.1	0.0	
**Alfios**	2.9	0.2	0.2	0.3
**RAG1**				
	**Evrotas**	**Pamissos**	**Miras**	**Alfios**
**Evrotas**	0.0			
**Pamissos**	1.0	0.1		
**Miras**	1.0	0.2	0.1	
**Alfios**	1.0	0.1	0.2	0.1

**Fig 7:**
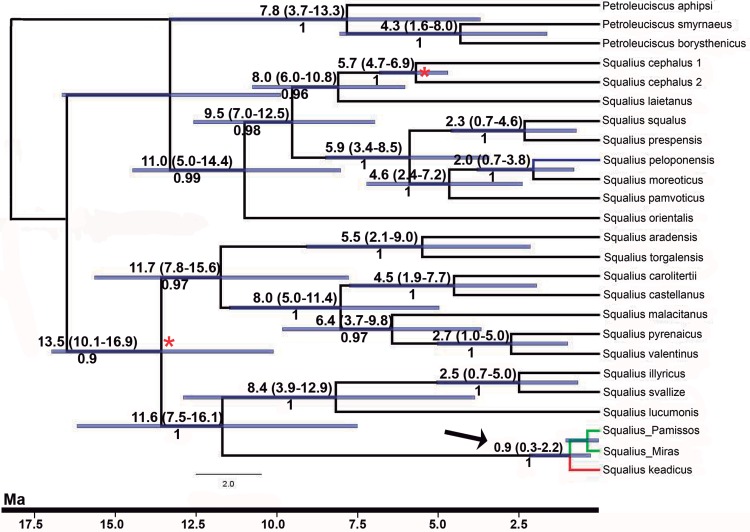
Chronogram obtained for the *MT-CYB* gene for several *Squalius* species. Divergence time estimates and their HPD 95% confidence intervals, in Ma, for the main cladogenetic events between *Squalius* species above branches. Posterior probability values of Bayesian inference below branches (** indicates posterior probability = 1). Black arrow highlights the divergence time estimated for the separation of mitochondrial lineages of *S*. *keadicus* and the introgressed populations. Red asterisk indicates the position of fossil ages used to calibrate the molecular clock.

Estimated divergence times of the mitochondrial lineage of *Squalius keadicus* from that of the introgressed populations of *S*. *peloponensis* was approximately 0.9 Ma (CI 95% HPD: 0.3–2.2 Ma; Fig 7). The divergence of *S*. *peloponensis* from closely related *Squalius* spp occurred in Middle-Upper Pliocene. However, this phylogenetic relationship was not highly supported, probably as a consequence of an incomplete sampling of the Euroasiatic lineage of this genus *sensu* [35] (see also [37]).

### Morphological analyses

**Fig 8:**
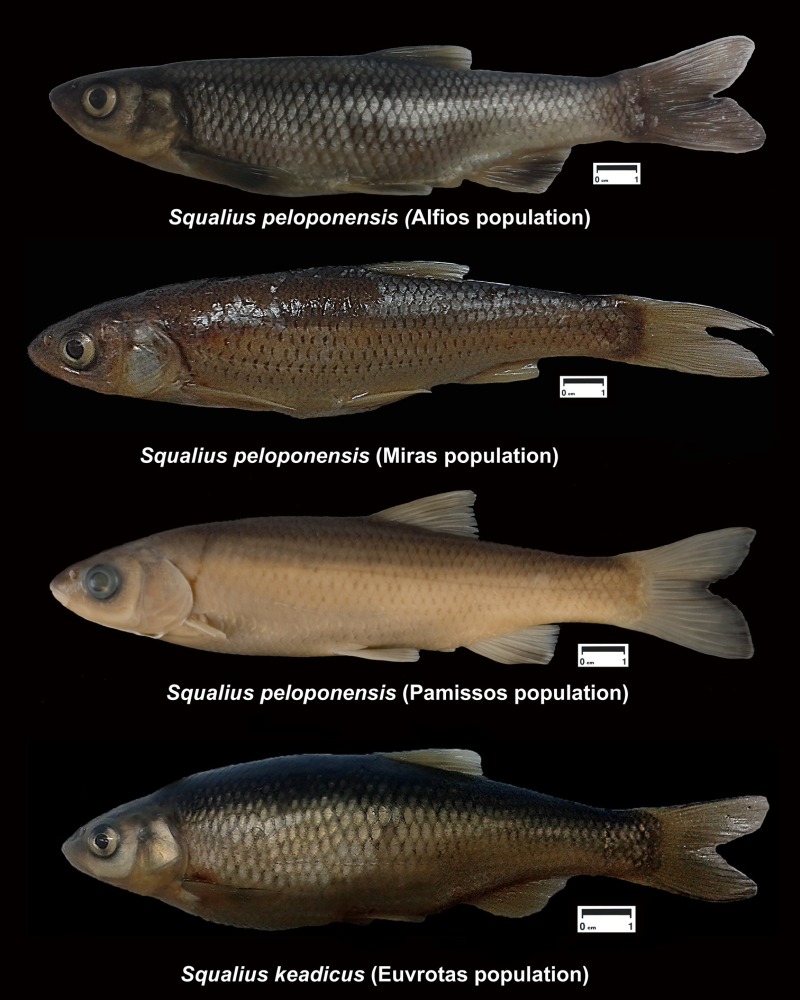
Specimens of the four analysed populations. Voucher specimen numbers: *Squalius peloponensis* from Alfios River (MNCN_ICTIO 94.982); *Squalius peloponensis* from Pamissos River (NMP PV6 F1227); *Squalius peloponensis* from Miras River (MNCN_ICTIO 94.747); *Squalius keadicus* from Evrotas River (MNCN_ICTIO 123.875).

**Fig 9:**
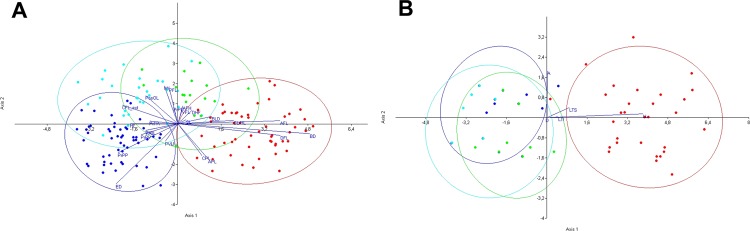
Canonical Variate Analysis of the four *Squalius* populations. A) Morphometric measurements. B) Meristic measurements. Red: Evrotas population (*S*. *keadicus*), blue: Alfios population (*S*. *peloponensis*); light blue: Miras population (*S*. *peloponensis*); green: Pamissos population (*S*. *peloponensis*).

Kruskal–Wallis and Mann–Whitney *post hoc* analyses demonstrated significant differences among the four analysed *Squalius* populations in morphometric and meristic variables (Table 3; Fig 8). Canonical variate analysis (CVA) also supported morphological differences, showing four isolated groups with partial overlap between the populations from the Miras and Pamissos basins and between each of these populations with both the Evrotas (*S*. *keadicus*) and Alfios (*S*. *peloponensis*) populations. As expected, overlap between *S*. *keadicus* and the Alfios *S*. *peloponensis* population was not observed (Fig 9). The most contributory morphometric variables to the ordination in the CVA were AFL, BD, and DFL (Table 4).

**Table 3:** Kruskal-Wallis test and non-parametric Mann-Whitney pairwise *post hoc* comparisons for all populations. Ev = Evrotas, Al = Alfios, Pa = Pamissos, Mi = Miras, Significant differences *p* < 0.01. Acronyms of variables are defined in the Material and Methods section.

Variable	H	P-value	Significant Mann-Whitney pairwise comparisons
SL	25.57	<0.0001	Al-Ev; Al-Pa
HL	78.95	<0.0001	Al-Ev; Al-Pa; Ev-Mi; Ev-Pa
ED	63.72	<0.0001	Al-Ev; Al-Pa; Al-Mi
IW	56.48	<0.0001	Al-Ev; Al-Pa; Al-Mi; Ev-Pa;
PrOL	19.51	0.0002	Al-Ev
PosOL	30.77	<0.0001	Al-Ev; Pa-Ev; Mi-Ev
PrPD	11.38	0.0099	-
PrPP	92.4	<0.0001	Al-Ev; Al-Pa; Al-Mi; Ev-Mi; Pa-Mi
PrPV	86.22	<0.0001	Al-Ev; Al-Pa; Al-Mi; Ev-Mi; Ev-Pa
PrPA	59.86	<0.0001	Ev-Al; Ev-Pa; Ev-Mi
DHF	13.39	0.0039	Al-Ev
DFL	84.98	<0.0001	Ev-Al; Ev-Pa; Ev-Mi; Al-Pa
CPL	16.06	0.0011	Ev-Mi
CFL	46.87	<0.0001	Ev-Al; Ev-Mi
BLD	16.6	0.0009	Ev-Al; Ev-Mi
BD	100.8	<0.0001	Ev-Al; Ev-Pa; Ev-Mi; Al-Pa
APL	21.72	<0.0001	Ev-Mi
AFL	56.89	<0.0001	Al-Ev; Al-Pa; Ev-Mi
AFH	7.91	0.0479	-
VAL	17.37	0.0006	Al-Ev
PVL	4.794	0.1875	-
VFL	10.42	0.0154	Al-Mi
PFL	11.64	0.0087	Mi-Al; Mi-Ev
LL	106.6	<0.0001	Ev-Al; Ev-Pa; Ev-Mi
UTS	74.29	<0.0001	Ev-Al; Ev-Pa; Ev-Mi; Al-Mi
LTS	40.74	<0.0001	Ev-Al; Ev-Pa; Ev-Mi
D	0.5093	0.9169	-
A	19.1	0.0002	Al-Pa; Al-Mi

**Table 4:** Canonical Variate Analysis values for the first three canonical axes of analyses of morphometric and meristic variables. Acronyms of variables are defined in the Material and Method section.

VARIABLE	AXIS 1	AXIS 2	AXIS 3
Eigenvalue 1 = 4.861 (77.76%)			
Eigenvalue 2 = 0.9997 (15.99%)			
SL	0.0009	0.0008	0.0028
HL	-0.0055	0.0003	0.0002
ED	-0.0069	-0.0094	0.0031
IW	-0.0016	0.0060	-0.0002
PrOL	-0.0038	-0.0014	-0.0049
PosOL	-0.0037	0.0048	0.0015
PrPD	-0.0005	0.0029	0.0003
PrPP	-0.0069	-0.0043	-0.0046
PrPV	-0.0042	-0.0015	0.0013
PrPA	-0.0032	0.0006	0.0018
DFH	0.0017	0.0029	-0.0030
DFL	0.0119	-0.0015	-0.0075
CPL	0.0027	-0.0051	0.0019
CFL	-0.0068	0.0021	-0.0028
BLD	0.0040	0.0008	0.0029
BD	0.0153	-0.0009	-0.0042
APL	0.0032	-0.0046	0.0085
AFL	0.0123	-0.0002	0.0070
AFH	0.0006	0.0032	-0.0044
VAL	-0.0037	-0.0012	0.0056
VFL	0.0004	0.0026	-0.0064
PVL	-0.0014	-0.0033	-0.0014
PFL	-0.0009	0.0051	-0.0028
Eigenvalue 1 = 8.176 (97.3%)			
Eigenvalue 2 = 0.21 (2.5%)			
LL	0.9484	0.0370	-0.0734
UTS	0.2201	0.0981	0.4004
LTS	0.1131	0.0053	-0.1368
D	-0.0126	-0.0023	0.0229
A	0.0051	0.4609	-0.0231

**Fig 10:**
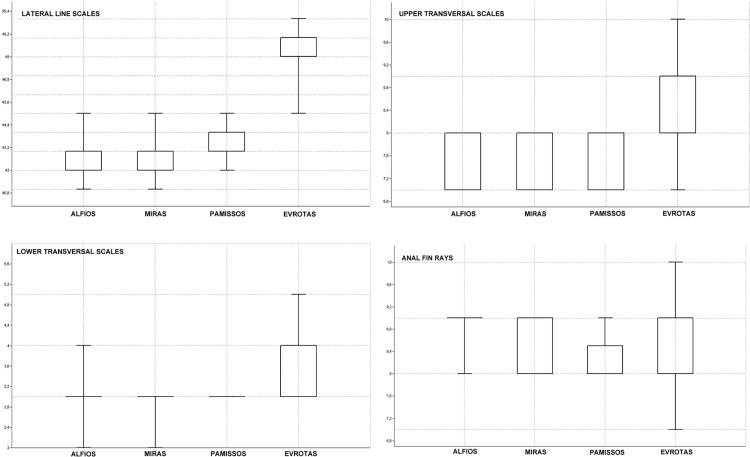
Box-plot of meristic variables. The 25–75 percent quartiles are drawn inside the box. Short horizontal lines represent minimal and maximal values.

Meristic variables in the Alfios, Pamissos, and Miras populations overlapped with the Evrotas population (*S*. *keadicus*) forming a differentiated group in CVA (Fig 9). Kruskal–Wallis and Mann–Whitney *post hoc* comparisons were significant for LL, LTS, LTI, and A, reaching higher values in *S*. *keadicus* relative to the other three populations (Fig 10).

The Evrotas population showed greater BD and longer APL, DFL and AFL fins than the other three populations. It also exhibited significantly shorter HL relative to SL than in other populations (Table 5). The Pamissos population exhibited the significantly smaller ED relative to HL and SL than seen in the other three populations. The largest ED relative to the standard length was found in the *S*. *peloponensis* from Alfios (Table 5). Although the raw data matrix was corrected by Burnaby’s method, differences in ED may be attributed to allometric growth rather than to real population differences. The Alfios population had the narrowest head in relation to HL (Table 5), a ratio significantly different from the Evrotas and Miras populations, but not from that of the Pamissos.

**Table 5:** Significant proportions (%) relative to standard length (SL) or head length (HL) of several morphometric variables differentiating the four analysed populations.

VARIABLE	POPULATION	% RELATIVE TO SL OR TO HL
BD relative to SL	Evrotas	25.9 (24.0–28.7)
	Alfios	21.6 (21.2–21.5
	Miras	22.4 (21.1–22.7)
	Pamissos	22.5 (23.0–24.7)
DFL relative to SL	Evrotas	12.6 (12.0–13.4)
	Alfios	11.1 (10.4–11.4)
	Miras	11.3 (10.5–11.4)
	Pamissos	11.5 (10.8–12.0)
AFL relative to SL	Evrotas	11.4 (8.9–12.6)
	Alfios	10.4 (8.7–12.3)
	Miras	10.2 (9.3–10.0)
	Pamissos	11.5 (10.5–12.7)
APL relative to SL	Evrotas	21.4 (21.0–21.7)
	Alfios	20.8 (20.9–21.1)
	Miras	20.2 (18.0–18.9)
	Pamissos	20.5 (19.9–19.9)
HL relative to SL	Evrotas	23.5 (24.4–24.6)
	Alfios	25.1 (24.9–26.3)
	Miras	24.9 (23.5–26.8)
	Pamissos	23.8 (23.6–26.0)
ED relative to HL	Evrotas	24.6 (24.0–25.5)
	Alfios	27.0 (24.8–28.9)
	Miras	27.0 (24.4–29.5)
	Pamissos	24.6 (24.0–25.5)
ED relative to SL	Evrotas	6.3 (6.0–7.2)
	Alfios	6.8 (6.2–7.6)
	Miras	6.5 (5.5–6.6)
	Pamissos	5.8 (5.9–6.6)
IW relative to HL	Evrotas	42.5 (39.2–40.2)
	Alfios	36.0 (35.4–39.6)
	Miras	42.0 (39.7–41.6)
	Pamissos	41.6 (41.2–41.9)

## Discussion

In this study we tested through morphological and molecular analyses the hypothesis of mitochondrial introgression between two species of the genus *Squalius* from Greece, *S*. *keadicus* and *S*. *peloponensis*, formulated on the basis of the discordance between mitochondrial (*MT-CYB*; [34]) and nuclear (allozymes; [33]) genomes found in one population from the Greek Peloponnese (from Miras Basin).

The populations from the Miras and Pamissos basins were morphologically similar to *S*. *peloponensis* from Alfios Basin, as expected based on sharing of the nuclear genome and the lack of diagnostic nuclear autapomorphies among the three populations (Table C in S1 File), as well as on meristic characters, which fall within the range of *S*. *peloponensis* (Fig 10). However, conserved meristic measurements are observed in *Squalius* species of the Euroasiatic lineage *sensu* [35] [37]. Although the populations from Miras and Pamissos were recognized as *S*. *peloponensis* significant differences between these two populations were found in some morphometric measurements, as well as between *S*. *peloponensis* and *S*. *keadicus* (Tables 3 and 5; Fig 9).

Whereas the Miras and Pamissos populations showed the nuclear genome and morphology typical of *Squalius peloponensis*, their mitochondrial genome clustered with *S*. *keadicus* (Evrotas population), but not with the *S*. *peloponensis* (Alfios population). This mitonuclear discordance supports the idea of mitochondrial introgression of *S*. *keadicus* in the Miras and Pamissos populations. Sharing of similar morphology and the nuclear genome allowed us to reject the hypothesis of Miras and Pamissos populations being hybrids of *S*. *keadicus* and *S*. *peloponensis*, since they should show similarity to both hypothetical parent species in the nuclear genome as well as in the mitochondrial genome. Therefore, the hypothesis of mitochondrial DNA introgression cannot be rejected based on our results. Hybridization is common in fish, and the boundaries between species may be obscure. In cyprinids, hybridization is a common evolutionary process [26, 66–69]. In our analyses, the two nuclear markers were *S*. *peloponensis*-like in introgressed populations, and we did not find evidence of recombination between species (PHI = 1 for both nuclear markers), as demonstrated by the phylogenetic relationships of phased alleles (Fig 6), and low (0.1–0.2%) genetic distance between populations in both nuclear markers. Hence, we accept the hypothesis of mitochondrial introgression and reject the hypothesis of hybrid speciation.

All analysed specimens from Miras and Pamissos were mitochondrially *S*. *keadicus*-like and nuclearly *S*. *peloponensis*-like, suggesting unidirectional complete mitochondrial introgression (mitochondrial capture) of *S*. *keadicus* to *S*. *peloponensis* [34]. Nonetheless, they formed an independent highly supported phylogenetic clade, sister to *S*. *keadicus* from the Evrotas population (Fig 3). These two highly supported reciprocal monophyletic groups found in the mitochondrial tree within the phylogenetic lineage of *S*. *keadicus* indicate that the introgression event was ancient, but it is not clear whether divergence of these populations, allowing mitochondrial DNA to sort, occurred before or after the introgression event. Ancient mitochondrial captures have also been described for reptiles [70–71], insects [18], and fishes [27–28]. Since these four *Squalius* populations currently occur in allopatry, a geographical barrier may be considered the primary source of their morphological differences possibly through a process of incipient speciation.

Isolation of populations and incipient speciation in sympatry can be attributed to sexual selection [72–73], divergent selection, and phenotypic plasticity [74], or segregation of ecological niches [75–76]. The morphological and molecular differentiation found among the analysed populations of *Squalius* from western Peloponnese probably occurred after their allopatric separation linked to the formation of the hydrological network in the region during the Upper Pliocene-Pleistocene periods [38, 43]. Incipient speciation or occurrence of differentiated lineages during the Pleistocene as a consequence of geographic barriers has been suggested for several taxa [77–79]. Examples of isolation-driven divergence to new lineages after ancient hybridization have been reported [19, 80]. This is demonstrated here by the lack of shared mitochondrial haplotypes among populations in the rivers of the western Peloponnese. The sharing of the nuclear genome among the Alfios, Miras, and Pamissos populations ascribed to *S*. *peloponensis*, clustering in the same clade, also supports the scenario of incipient speciation.

**Fig 11:**
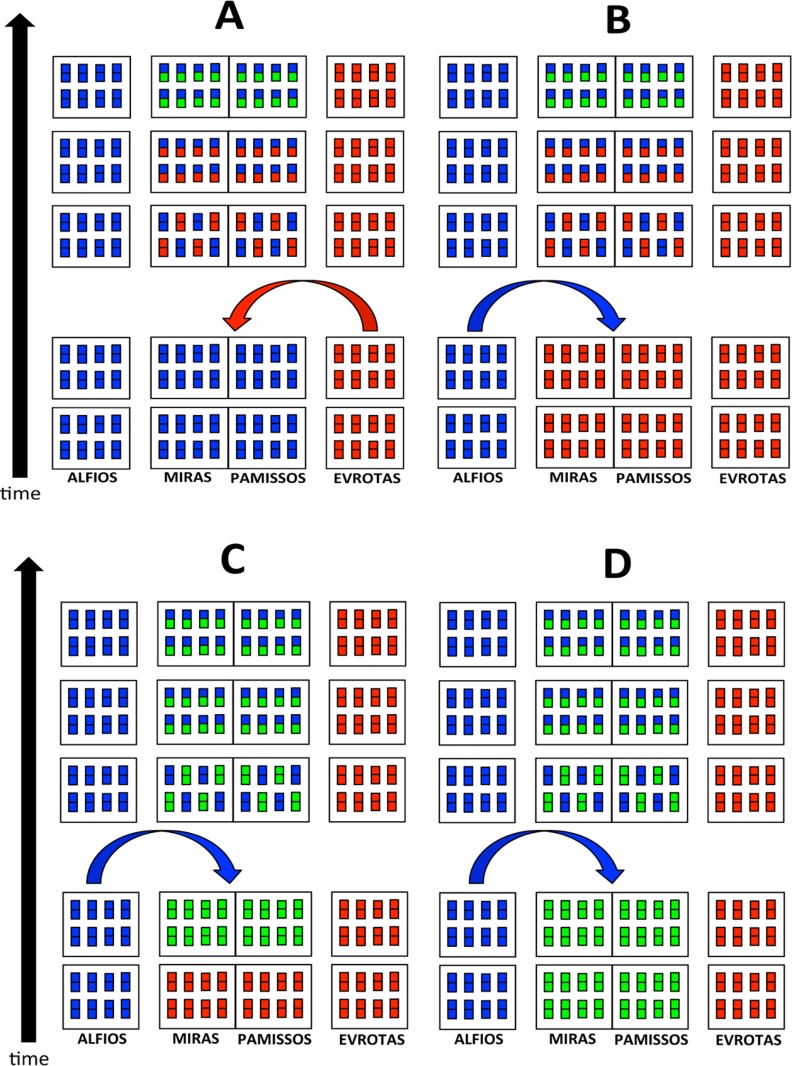
Hypothetical biogeographical scenarios explaining the mitochondrial capture event from *Squalius keadicus* to *Squalius peloponensis*. Each rectangle symbolizes a hypothetical individual within the analysed population. The two squares within each rectangle symbolize the mitochondrial and the nuclear genome of this hypothetical individual in the population. Colours indicate populations: Blue = *S*. *peloponensis* lineage; Red = *S*. *keadicus* lineage; Green = *S*. *keadicus*-like mitochondrial lineage (Miras and Pamissos populations).

Several biogeographic scenarios may be proposed to explain the mitochondrial introgression event and the current molecular configuration of the four analysed populations (Fig 11): A) The lineage of *Squalius peloponensis* inhabited the Alfios, Miras, and Pamissos basins, and at some time the lineage of *S*. *keadicus* invaded the Miras and Pamissos basins. The introgression took place, and subsequently mitogenomes of the mitochondrial lineages of *S*. *keadicus* from the Evrotas basin and the introgressed populations of the Miras and Pamissos diverged. B) The lineage of *Squalius keadicus* inhabited the Evrotas, Miras, and Pamissos basins, and at some time the lineage of *S*. *peloponensis* invaded the Miras and Pamissos basins. The introgression event took place, mitogenomes of the lineages of *S*. *keadicus* from the Evrotas basins and the introgressed Miras and Pamissos populations subsequently diverged. C) Miras and Pamissos basins were inhabited by the lineage of *Squalius keadicus* and the mitogenome of these populations diverged from that of the Evrotas population. The lineage of *S*. *peloponensis* then invaded the Miras and Pamissos basins and introgression occurred. D) A case similar to scenario C is that in which it was not the mitochondrial lineage of *Squalius keadicus* that inhabited the Miras and Pamissos basins prior to the invasion by the lineage of *S*. *peloponensis*, but a phylogenetically closely related species.

The main difference in these biogeographical scenarios is the timing of the introgression event as before (scenarios A and B) or after (scenarios C and D) the divergence of the *S*. *keadicus* from the *S*. *keadicus*-like mitogenome. The common factor among the hypothetical scenarios is the biogeographical discordance between mitochondrial and nuclear DNA [4]. Most patterns of biogeographic mitonuclear discordance are characterized by previously isolated lineages that came into contact and generated hybrid zones in which gene flow patterns in mitochondrial and nuclear DNA differed [4]. This hypothesis of secondary contact is consistent with the hypothetical biogeographical scenarios that we propose, which differ only whether the invader species is *S*. *keadicus* (A) or *S*. *peloponensis* (B, C, and D). Differences in effective population size of the resident and the invader species could be responsible for the unidirectionality of the introgression event found in the present study [2, 4, 81]. In the initial stages of an invasion event, a small number of invader specimens are expected in comparison to the resident population, and simulated studies have revealed unidirectional introgression of neutral loci from the resident to the invader population [21]. This would imply that *S*. *keadicus* (B, C) or *S*. *keadicus*-like (D) was the resident species and *S*. *peloponensis* the invader. Nevertheless, if the invader becomes more numerous as invasion continues, the direction of the introgression will depend of the demographic balance of the resident and invader populations [21, 82]. Mitochondrial DNA introgression as a consequence of extreme differences in population size has been described in other freshwater fish species [75]. In our hypothetical scenarios, *S*. *keadicus* or an extinct species phylogenetically similar to *S*. *keadicus*, regardless of whether it was the resident or the invader species, must have had a population large enough to erode the mitochondrial genome of *S*. *peloponensis* to the point of elimination. Another possible source of mitonuclear discordance may be sex-biased reproduction or sex-biased offspring production during the hybridization event [4]. Empirical experiments are necessary to assess this hypothesis.

Differences in distribution range of two species involved in an introgression event when a hybrid zone is formed explain asymmetric mitochondrial introgression, associated with differences in population size and competition between their mitochondrial genomes [4]. It is not possible to estimate the range of *S*. *keadicus* or the size of the hybrid zone in the Miras and Pamissos basins, because *S*. *keadicus*, or the *S*. *keadicus*-like species, is currently extinct in this region and no fossil evidence exists, but it is clear that the distribution range of the studied species overlapped in the past.

Although the mitochondrial genome is generally considered to be neutral [11], positive mitochondrial selection has been described for this marker [20], and is another possible explanation of the observed unidirectional mitochondrial introgression. Thus, the mitochondrial genome with the highest fitness may be introgressed into the other species, regardless of whether it is resident or invader [81]. These selective sweeps have been attributed to climatic adaptation, especially to temperature [83]. Our study area, however, was restricted to a small geographical region with no significant climatic differences in river systems. Furthermore, the HKA test of selection [84] performed on the *MT-CYB* gene (p>0.05) suggested the potential for neutral evolution in this gene, and a selective sweep is not indicated in the studied species.

The most recent common ancestor of the mitochondrion of the Evrotas *S*. *keadicus* and the introgressed populations of the Miras and Pamissos basins dated from 0.9 Ma (CI 95% HPD 0.3–2.2 Ma) in the Pleistocene, and introgression may have occurred before or after divergence. Mitochondrial captures during Pleistocene have been described for other freshwater fish species [28]. The divergence of the mitochondrial clades in the present study may be attributed to Pleistocene tectonic movements related to uplift and river system formation. The paleogeographical history of western Greece and the Peloponnese is complex; the area comprises a network of active faults that constitutes a multifractured neotectonic macrostructure [38]. Tectonic movements occurred throughout the Upper Pliocene-Pleistocene period and continue to the present causing earthquakes and tsunamis [39, 85]. The faults had great influence on the drainage network formation [38]. The current hydrological basins of the western Peloponnese were formed during the Pleistocene as a consequence of marine or continental sediment infilling when fault systems were activated. Marine sediments found at several hundred metres elevation in some mountainous areas are evidence of vertical tectonic movements throughout this period [38, 86]. These sedimentary events have contributed to configuration of the main fluvial basins of western Peloponnese, which show a radial pattern commonly found in areas of rapid uplifting [43].

The recent paleogeographic events may be responsible for the divergence of mitochondrial lineages of *S*. *keadicus* and the introgressed populations (mitochondrial *S*. *keadicus*–like) of *S*. *peloponensis* as well as morphological differences among the four populations that evolved following the isolation of the hydrological basins (Fig 11). Complex tectonic movements configuring the hydrological network of western Peloponnese may also have been the source of contact between species and the introgression event, through river piracy.

## Conclusions

Molecular and morphological evidence supports the hypothesis of mitochondrial introgression of *S*. *keadicus* to *S*. *peloponensis*. We found unidirectional mitochondrial introgression from *S*. *keadicus* into populations from two basins of the western Peloponnese, the Miras and Pamissos River systems, but not into that from the Alfios Basin, and a sorting of the mitochondrial genome of introgressed populations from that of *S*. *keadicus*, likely as a consequence of isolation by biogeographical barriers, leading to a process of incipient speciation. Recent secondary contact among basins is the most plausible hypothesis to explain the mitochondrial introgression.

The identification of mitochondrial capture is crucial to understanding the evolutionary history of living organisms, and adequate molecular and morphological analyses are essential to distinguish among evolutionary processes leading to mitonuclear discordance, such as the case of mitochondrial introgression.

## Supporting Information

S1 File Table A. Additional species used in phylogenetic performance. GenBank accession numbers and labels in phylogenetic trees. Table B. Evolutionary models estimated by jModelTest for the three analysed genes. Table C. Autapomorphies in the three analysed genes. * transversions.
(DOCX)
