# Supplementary material for: Ancient Mitochondrial Capture as Factor Promoting Mitonuclear Discordance in Freshwater Fishes: A Case Study in the Genus Squalius (Actinopterygii, Cyprinidae) in Greece
Source: PLoS One. 2016 Dec 1;11(12):e0166292. doi: 10.1371/journal.pone.0166292 (PMC5132402; doi:10.1371/journal.pone.0166292)
Supplement: S1 File — Table A. Additional species used in phylogenetic performance. GenBank accession numbers and labels in phylogenetic trees. Table B. Evolutionary models estimated by jModelTest for the three analysed genes. Table C. Autapomorphies in the three analysed genes. * transversions. (DOCX) [file pone.0166292.s001.docx]

Table A. Additional species used in phylogenetic performance. GenBank accession numbers and labels in phylogenetic trees.

| **Species** | **Locality** | **GenBank accession numbers (labels in phylogenetic trees)** |
| --- | --- | --- |
| *Petroleuciscus aphipsi* | Aphips River, Russia | *MT-CYB*: HM560177 (1), HM560178 (2)  *S7*: HM560528  *RAG1*: HM560455 |
| *Petroleuciscus borysthenicus* | Fotolivos, Greece | *MT-CYB*: HM560111  *S7*: HM560499  *RAG1*: HM560414 |
| *Petroleuciscus smyrnaeus* | Golcuk Lake, Turkey | *MT-CYB*: HM560114  *S7*: HM560501  *RAG1*: HM560416 |
| *Squalius aradensis* | Algibre R. Arade Basin, Portugal | *MT-CYB*: HM560179 |
| *Squalius carolitertii* | Limia River, Spain | *MT-CYB*: HM560182  *S7*: HM560529  *RAG1*: HM560446 |
| *Squalius castellanus* | Gallo River, Tajo Basin, Spain | *MT-CYB*: DQ521423  *S7*: HM560565  *RAG1*: HM560575 |
| *Squalius malacitanus* | Guadiaro River, Spain | *MT-CYB*: HM560192  *S7*: HM560531  *RAG1*: HM560449 |
| *Squalius pyrenaicus* | Guadalete River, Spain | *MT-CYB*: HM560198  *S7*: HM560568  *RAG1*: HM560579 |
| *Squalius valentinus* | Magro River, Jucar Basin, Spain | *MT-CYB*: HM560212  *S7*: HM560541  *RAG1*: HM560461 |
| *Squalius illyricus* | Cetina Basin, Croatia | *MT-CYB*: HM560183 (1), HM560184 (2)  *S7*: HM560530  *RAG1*: HM560447 |
| *Squalius svallize* | Rama Lake, Neretva Basin (1), Zalomka River, (2). Bosnia-Herzegovina | *MT-CYB*: HM560358 (1), HM560359 (2)  *S7*: HM560538  *RAG1*: HM560458 |
| *Squalius lucumonis* | Arno Basin, Italy | *MT-CYB*: HM560190  *RAG1*: HM560448 |
| *Squalius cephalus* | Elbe R. (1), Oder R. (2), Germany | *MT-CYB*: AJ252807 (1), AY549461 (2) |
| *Squalius laietanus* | Matarraña River, Spain | *MT-CYB*: HM560187, HM560188 |
| *Squalius squalus* | Vipava R. Isonzo Basin. Slovenia (1), Arno River, Italy (2) | *MT-CYB*: HM560204 (1), HM560205 (2)  *S7*: HM560537  *RAG1*: HM560457 |
| *Squalius prespensis* | Prespa Lake, Greece | *MT-CYB*: AF090753 (1), HM560197 (2)  *S7*: HM560564  *RAG1*: HM560457 |
| *Squalius orientalis* | Uluchaj River, Russia | *MT-CYB*: HM560195 (1), HM560196 (2)  *S7*: HM560533  *RAG1*: HM560451 |
| *Squalius moreoticus* | Stymphalia Lake, Greece | *MT-CYB*: KY070375-KY070380 (1-6) |
| *Squalius pamvoticus* | Arachthos River (1-3), Louros River (4-6), Greece | *MT-CYB*: KY070381-KY070383 (1-3), KY070384-KY070386 (4-6)  *S7*: KY070513 (1a), KY070514 (1b) |

Table B. Evolutionary models estimated by jModelTest for the three analysed genes

| **GENE** | **SELECTED MODEL** | **FREQUENCY OF BASES** | **RATE MATRIX** | **GAMMA SHAPE / INVARIABLE SITES** |
| --- | --- | --- | --- | --- |
| *MT-CYB* | TIM3+I+G  -lnL=5969.3629  k=184 | A = 0.2729  C = 0.2970  G = 0.1513  T = 0.2789 | R(a) [AC] = 1.9146  R(b) [AG] = 66.5160  R(c) [AT] = 1.0000  R(d) [CG] = 1.9146  R(e) [CT] = 13.8820  R(f) [GT] = 1.0000 | Gamma shape = 2.1360  Proportion of invariable sites = 0.5940 |
| *S7* | TPM3uf+G  -lnL=2394.4522  k=116 | A = 0.2943  C = 0.1545  G = 0.1982  T = 0.3530 | R(a) [AC] = 1.5458  R(b) [AG] = 2.8400  R(c) [AT] = 1.0000  R(d) [CG] = 1.5458  R(e) [CT] = 2.8400  R(f) [GT] =1.0000 | Gamma shape = 0.3030 |
| *RAG1* | TIM2ef+G  -lnL=2816.9850  k=116 | Equal | R(a) [AC] =  R(b) [AG] =  R(c) [AT] =  R(d) [CG] =  R(e) [CT] =  R(f) [GT] = | Gamma shape = 0.0720 |

Table C. Autapomorphies in the three analysed genes. * transversions

| **POSITION *MT-CYB*/**  **POPULATION** | 18 | 42 | 45 | 60 | 66 | 81 | 88 * | 93 | 105 | 108 | 117 | 144 | 150 | 151 | 156 | 159 | 162 | 174 | 180 * | 195 * | 204 | 228 * | 231 | 237 | 261 * | 273 | 279 | 285 |
| --- | --- | --- | --- | --- | --- | --- | --- | --- | --- | --- | --- | --- | --- | --- | --- | --- | --- | --- | --- | --- | --- | --- | --- | --- | --- | --- | --- | --- |
| ALFIOS | **A** | **C** | **T** | **C** | **G** | **C** | **C** | **G** | **G** | **C** | A | **G** | **C** | **C** | **G** | **G** | **C** | **C** | **G** | **G** | **C** | **C** | **G** | **T** | **C** | **T** | **C** | **C** |
| EVROTAS | G | T | C | T | A | T | A | A | C | T | **G** | A | T | T | A | A | T | T | C | C | T | A | A | C | A | C | T | T |
| MIRAS | G | T | C | T | A | T | A | A | C | T | A | A | T | T | A | A | T | T | C | C | T | A | A | C | A | C | T | T |
| PAMISSOS | G | T | C | T | A | T | A | A | C | T | A | A | T | T | A | A | T | T | C | C | T | A | A | C | A | C | T | T |

…. Continuation Table C

| **POSITION *MT-CYB*/**  **POPULATION** | 291 | 303 * | 309 | 315 | 327 | 351 | 352 | 366 | 372 | 378 | 384 | 390 | 393 | 396 | 405 | 411 | 426 | 432 | 441 | 444 | 462 | 465 | 468 | 474 | 495 | 510 | 513 | 519 |
| --- | --- | --- | --- | --- | --- | --- | --- | --- | --- | --- | --- | --- | --- | --- | --- | --- | --- | --- | --- | --- | --- | --- | --- | --- | --- | --- | --- | --- |
| ALFIOS | **C** | **A** | **C** | **T** | **T** | **A** | **A** | **A** | **A** | **A** | **A** | **T** | **C** | **A** | **A** | **A** | **T** | **G** | **A** | **C** | **T** | **C** | **A** | C | **A** | **A** | **T** | **G** |
| EVROTAS | T | C | T | C | C | G | G | G | G | G | G | C | T | G | G | G | C | A | G | T | C | T | G | C | G | G | C | A |
| MIRAS | T | C | T | C | C | A | G | G | G | G | G | C | T | G | G | G | C | A | G | T | C | T | G | C | G | G | C | A |
| PAMISSOS | T | C | T | C | C | A | G | G | G | G | G | C | T | G | G | G | C | A | G | T | C | T | G | **T** | G | G | C | A |

…. Continuation Table C

| **POSITION *MT-CYB*/**  **POPULATION** | 522 | 552 | 573 | 577 | 579 * | 580 | 582 | 585 * | 592 | 598 | 606 | 609 | 615 | 630 * | 639 | 642 | 651 | 675 * | 693 * | 696 | 700 | 702 * | 708 | 709 | 717 * | 723 | 724 | 748 |
| --- | --- | --- | --- | --- | --- | --- | --- | --- | --- | --- | --- | --- | --- | --- | --- | --- | --- | --- | --- | --- | --- | --- | --- | --- | --- | --- | --- | --- |
| ALFIOS | **C** | **C** | **T** | A | **C** | **A** | **C** | **A** | **C** | **C** | **G** | **G** | **A** | **C** | **C** | **T** | **A** | **A** | T | **C** | **A** | **C** | **G** | **T** | **T** | **C** | **T** | **C** |
| EVROTAS | T | A | C | **G** | G | G | T | C | T | T | A | A | G | A | T | C | G | C | **C** | T | G | A | A | C | A | T | C | T |
| MIRAS | T | A | C | A | G | G | T | C | T | T | A | A | G | A | T | C | G | C | A | T | G | A | A | C | A | T | C | T |
| PAMISSOS | T | A | C | A | G | G | T | C | T | T | A | A | G | A | T | C | G | C | A | T | G | A | A | C | A | T | C | T |

…. Continuation Table C

| **POSITION *MT-CYB*/**  **POPULATION** | 750 | 759 | 774 * | 777 | 784 | 786 | 789 | 792 | 798 | 813 | 816 | 819 | 840 | 849 | 852 | 858 | 861 | 888 | 894 | 897 | 900 | 906 | 915 | 918 | 930 |
| --- | --- | --- | --- | --- | --- | --- | --- | --- | --- | --- | --- | --- | --- | --- | --- | --- | --- | --- | --- | --- | --- | --- | --- | --- | --- |
| ALFIOS | **G** | **G** | **A** | **A** | **C** | **G** | **G** | **T** | **A** | **G** | **G** | **T** | **T** | **T** | **C** | **C** | **A** | **C** | **C** | **A** | **G** | **G** | **A** | **C** | **T** |
| EVROTAS | A | A | T | G | T | A | A | **G** | G | A | A | C | C | C | T | T | G | T | T | G | A | A | C | T | G |
| MIRAS | A | A | T | G | T | A | A | A | G | A | A | C | C | C | T | T | G | T | T | G | A | A | C | T | G |
| PAMISSOS | A | A | T | G | T | A | A | A | G | A | A | C | C | C | T | T | G | T | T | G | A | A | C | T | G |

…. Continuation Table C

| **POSITION *MT-CYB*/**  **POPULATION** | 1005 | 1017 | 1057 | 1069 | 1074 | 1078 | 1083 | 1089 | 1090 | 1092 | 1095 | 1110 | 1125 | 1129 |
| --- | --- | --- | --- | --- | --- | --- | --- | --- | --- | --- | --- | --- | --- | --- |
| ALFIOS | **G** | **G** | **G** | **C** | **T** | **G** | **C** | **C** | **A** | **T** | **T** | **A** | **A** | **C** |
| EVROTAS | A | A | A | T | C | A | A | A | G | C | C | C | G | T |
| MIRAS | A | A | A | T | C | A | A | A | G | C | C | C | G | T |
| PAMISSOS | A | A | A | T | C | A | A | A | G | C | C | C | G | T |

…. Continuation Table C

| **POSITION *S7*/**  **POPULATION** | 68 * | 69 | 72 | 99 | 130 | 266 * | 313 | 414-419 | 463-477 | 530 | 565 | 581 * | 608 * | 639 |
| --- | --- | --- | --- | --- | --- | --- | --- | --- | --- | --- | --- | --- | --- | --- |
| ALFIOS | A | C | C | C | T | T | A | ACGTT | - | G | A | A | A | T |
| EVROTAS | **T** | **T** | **T** | **T** | **C** | **A** | **G** | **-** | **TGAGATTAATAATTT** | **A** | **G** | **T** | **C** | **C** |
| MIRAS | A | C | C | C | T | T | A | ACGTT | - | G | A | A | A | T |
| PAMISSOS | A | C | C | C | T | T | A | ACGTT | - | G | A | A | A | T |

…. Continuation Table C

| **POSITION *S7*/**  **POPULATION** | 642-645 | 647 | 653 | 684 * | 732 * | 751-752 | 754 | 759 | 802 * | 819 * | 829 | 855 * | 857 | 870 | 876 | 877-892 | 945-946 |
| --- | --- | --- | --- | --- | --- | --- | --- | --- | --- | --- | --- | --- | --- | --- | --- | --- | --- |
| ALFIOS | - | - | T | A | A | TT | T | T | A | G | A | A | G | A | G | - | TG |
| EVROTAS | **TTA** | **G** | **-** | **T** | **T** | **-** | **C** | **C** | **C** | **T** | **G** | **T** | **A** | **G** | **A** | **ACACTATTTTAAGTG** | **-** |
| MIRAS | - | - | T | A | A | TT | T | T | A | G | A | A | G | A | G | T | TG |
| PAMISSOS | - | - | T | A | A | TT | T | T | A | G | A | A | G | A | G | T | TG |

…. Continuation Table S2

| **POSITION *RAG1*/**  **POPULATION** | 439 | 768 * | 773 | 898 | 948 * | 1002 | 1008 | 1026 | 1032 | 1048 * | 1161 * | 1169 | 1173 |
| --- | --- | --- | --- | --- | --- | --- | --- | --- | --- | --- | --- | --- | --- |
| ALFIOS | G | T | A | T | C | G | C | G | G | C | T | A | G |
| EVROTAS | **A** | **A** | **G** | **C** | **A** | **A** | **T** | **A** | **A** | **A** | **A** | **G** | **A** |
| MIRAS | G | T | A | T | C | G | C | G | G | C | T | A | G |
| PAMISSOS | G | T | A | T | C | G | C | G | G | C | T | A | G |
